# Expression of chemokines CXCL4 and CXCL7 by synovial macrophages defines an early stage of rheumatoid arthritis

**DOI:** 10.1136/annrheumdis-2014-206921

**Published:** 2015-04-09

**Authors:** L Yeo, N Adlard, M Biehl, M Juarez, T Smallie, M Snow, C D Buckley, K Raza, A Filer, D Scheel-Toellner

**Affiliations:** 1 Rheumatology Research Group, Centre for Translational Inflammation Research, College of Medical and Dental Sciences, University of Birmingham, Birmingham, UK; 2 Johann Bernoulli Institute for Mathematics and Computer Science, University of Groningen, University of Groningen, Groningen, The Netherlands; 3 Royal Orthopaedic Hospital NHS Foundation Trust, Birmingham, UK; 4 Sandwell and West Birmingham Hospitals NHS Trust, Birmingham, UK; 5 University Hospitals Birmingham NHS Foundation Trust, Birmingham, UK

**Keywords:** Cytokines, Early Rheumatoid Arthritis, Inflammation, Synovitis, Rheumatoid Arthritis

## Abstract

**Background and objectives:**

For our understanding of the pathogenesis of rheumatoid arthritis (RA), it is important to elucidate the mechanisms underlying early stages of synovitis. Here, synovial cytokine production was investigated in patients with very early arthritis.

**Methods:**

Synovial biopsies were obtained from patients with at least one clinically swollen joint within 12 weeks of symptom onset. At an 18-month follow-up visit, patients who went on to develop RA, or whose arthritis spontaneously resolved, were identified. Biopsies were also obtained from patients with RA with longer symptom duration (>12 weeks) and individuals with no clinically apparent inflammation. Synovial mRNA expression of 117 cytokines was quantified using PCR techniques and analysed using standard and novel methods of data analysis. Synovial tissue sections were stained for CXCL4, CXCL7, CD41, CD68 and von Willebrand factor.

**Results:**

A machine learning approach identified expression of mRNA for CXCL4 and CXCL7 as potentially important in the classification of early RA versus resolving arthritis. mRNA levels for these chemokines were significantly elevated in patients with early RA compared with uninflamed controls. Significantly increased CXCL4 and CXCL7 protein expression was observed in patients with early RA compared with those with resolving arthritis or longer established disease. CXCL4 and CXCL7 co-localised with blood vessels, platelets and CD68^+^ macrophages. Extravascular CXCL7 expression was significantly higher in patients with very early RA compared with longer duration RA or resolving arthritis

**Conclusions:**

Taken together, these observations suggest a transient increase in synovial CXCL4 and CXCL7 levels in early RA.

## Introduction

The rheumatoid synovium is characterised by a complex inflammatory infiltrate, which can either be highly structured with distinct features of lymphoid neogenesis or comprise a more diffuse infiltrate. There is considerable evidence that cytokines produced by the synovial infiltrate play an important role in the orchestration of both the development and the resolution of synovial inflammation.^1^ Since there is evidence that therapeutic outcome in rheumatoid arthritis (RA) is influenced by the time elapsed before initiation of therapy, there is a considerable clinical need to diagnose patients with early disease.^2–4^ Due to the important role of cytokines in the regulation of the inflammatory infiltrate and the need to understand and diagnose early RA, this study systematically addresses the level of mRNA expression of a wide range of cytokines in the early stages of synovial inflammation.

In order to capture a population of patients in a time frame close to the onset of clinically apparent joint inflammation, patients with at least one clinically swollen joint were seen within 12 weeks of the onset of any symptom attributed, by the assessing rheumatologist, to an inflammatory arthritis. At this time, among a range of other investigations performed within the Birmingham Early Inflammatory Arthritis Cohort (BEACON), ultrasound-guided biopsies were taken. At an 18-month follow-up visit, patients who had progressed to RA as classified according to the 1987 American College of Rheumatology (ACR) criteria^5^ or had resolving disease were identified for this study. Furthermore, patients from the same clinic who were disease-modifying antirheumatic drug (DMARD) naive but had synovitis for >12 weeks, as well as patients attending a clinic due to mechanical joint problems without any clinically observed inflammation, were investigated as control groups.

Profiling of cytokine mRNA expression led to the finding that the chemokines CXCL4 and CXCL7 are expressed during the earliest phase of RA, but not in patients with resolving arthritis or established RA. We found that both chemokines, which are classically regarded as platelet-derived chemokines, were also expressed on macrophages in the synovium, implicating a previously undescribed role for this cell type in the earliest clinically evident stages of RA.

## Patients and methods

### Study participants

Patients with early arthritis were seen in the BEACON cohort; details of this clinic have been reported previously.^6^ Patients were eligible for the early arthritis cohort if they had at least one clinically swollen joint, were seen within 12 weeks of the onset of any symptom attributed by the assessing rheumatologist to an inflammatory arthritis and had not been treated with either DMARD or glucocorticoids prior to referral. Patients underwent a 68-joint clinical examination.^7^ Patients with a joint amenable to ultrasound-guided biopsy^8^ were recruited for this study. Following the biopsy, patients were followed for up to 18 months. Patients were identified for inclusion in the study if they were classified as having RA according to 1987 ACR criteria,^5^ or a resolving arthritis defined as the absence of clinically apparent synovial swelling at final assessment with no DMARDs or glucocorticoids having been used for the previous three months. A small number of patients developed chronic inflammatory diseases of the joint other than RA (table 1). As a control group, we included patients with RA with disease duration (defined as the time from the onset of any symptom attributed by the assessing rheumatologist to an inflammatory arthritis) of >12 weeks who fulfilled the 1987 ACR criteria^5^ at the time of biopsy. Similarly to patients with early arthritis, those with RA of >12 weeks’ duration were all DMARD and glucocorticoid naive and synovial tissue was obtained by ultrasound-guided biopsy. As a further control group, we included ‘uninflamed controls’ who underwent knee arthroscopy because of unexplained joint pain. None of these subjects showed inflammatory or degenerative joint pathology upon physical examination or arthroscopy. All researchers involved in the investigation of biopsies either at mRNA or protein level were blinded to patient outcome throughout the study.

**Table 1:** Demographic and clinical characteristics of study participants used for cytokine and chemokine mRNA real-time PCR low-density arrays

	Uninflamed	Resolving arthritis	Early RA	Established RA
Number	10	9	17	12
Symptom duration (weeks); median (IQR)	na	5 (2–9)	6 (4–9)	38 (27–52)
Female; n (%)	5 (50)	3 (33)	12 (71)	6 (50)
Age years; median (IQR)	43 (37–48)	40 (30–69)	53 (48–59)	60 (48–67)
RF and/or anti-CCP positive; n (%)	na	0 (0)	8 (47)	7 (58)
*Global disease-related variables*	** **	** **	** **	** **
CRP; median (IQR)	na	10 (8–22)	12 (5–32)	17 (7–52)
ESR; median (IQR)	na	24 (8–48)	25 (14–58)	28 (12–55)
DAS28; median (IQR)	na	4.1 (3.4–4.6)	4.7 (4.2–6.0)	5.4 (4.7–6.6)
*Biopsied joint-related variables*				
Joint biopsied				
Ankle; n (%)	0 (0)	3 (33.3)	4 (24)	2 (17)
Knee; n (%)	10 (100)	6 (66.6)	10 (59)	10 (83)
MCP joint; n (%)	0 (0)	0 (0)	3 (18)	0 (0)

CCP, cyclic citrullinated peptide; CRP, C-reactive protein; DAS28, Disease Activity Score in 28 Joints; ESR, erythrocyte sedimentation rate; MCP, metacarpophalangeal; na, not available; RF, rheumatoid factor.

### Ultrasound-guided synovial biopsy

Prior to biopsy, joints were assessed using a Siemens Acuson Antares scanner (Siemens, Bracknell, UK) and multifrequency (5–13 MHz) linear array transducers; for details, see Filer *et al*.^7^ Ultrasound-guided biopsy was used to collect tissue from multiple regions within knee, ankle or metacarpophalangeal (MCP) joints in which there was evidence of grey-scale synovitis. Ultrasound guidance was used to introduce a single portal through which tissue was sampled using custom manufactured 2.0 mm cutting-edged forceps or a 16 g core biopsy needle (MCP joint).^9^ Frozen blocks were assembled for processing from six individual biopsies in order to overcome synovial heterogeneity.^8^ ^10–12^

### Synovial tissue cytokine mRNA real-time PCR analysis

TaqMan low-density real-time PCR arrays (Applied Biosystems, Paisley, UK) were designed to determine expression of 117 cytokines and cytokine-related molecules (for full details, see online supplementary table S1). RNA was extracted from synovial tissue sections using an RNeasy RNA extraction kit (Qiagen, Crawley, UK). RNA from synovial tissue used in the low-density array for genes with non-intron spanning primers was treated with DNase (Qiagen). A reaction mixture containing RNA, QuantiTect-RT Master Mix (Qiagen) and QuantiTect Reverse Transcriptase (Qiagen, Crawley, UK) was added to a TaqMan low-density array microfluidic card. Six times more RNA was loaded into microfluidic cards designed for weakly expressed genes and genes with non-intron spanning primers (see online supplementary table S1). Reverse transcription and real-time PCR was performed in a 7900HT Real-Time PCR System (Applied Biosystems). Relative gene expression (RQ) was expressed as 2^−ΔCt^, where ΔCt represents the difference in Ct between glyceraldehyde-3-phosphate dehydrogenase and the target gene. Validation experiments established the reproducibility of quantitation and a combination of positive controls from anti-CD3/anti-CD28-activated lymphocytes and commercially available control mRNA from human cell lines representing different tissues (Stratagene) were used to ascertain that all cytokines listed could be detected.

### GMLVQ analysis of cytokine mRNA profiles

Cytokine mRNA data were log-transformed and all zero values were replaced by 0.00002, the smallest non-zero expression value in the data. This yielded 117 log-transformed expression values per patient. These data were analysed by applying a combination of principal component analysis and learning vector quantisation (LVQ), a distance-based classification technique, which determines class representatives from a set of example data in an iterative training process. Example data corresponded to the individual cytokine profiles observed within the four classes of patients. Matrix relevance LVQ identifies a suitable distance measure, which is discriminative with respect to the different classes. The specific technique used was generalised matrix relevance LVQ (GMLVQ), a variant that optimises the distance measure with respect to its discriminative power.^13^ Further details on this methodology can be found in online supplementary method 1.

### Immunofluorescence

Frozen sections from synovial biopsies taken from patients with resolving arthritis, early RA and established RA were stained with antibodies specific for CXCL4 (Abcam, UK) or CXCL7 (Novus Biologicals, UK), and CD41 (Dako, UK), CD68 (Thermo Scientific Pierce, UK) and von Willebrand factor (vWF) (Dako, UK). Staining with isotype-matched, species-matched and concentration-matched negative controls was performed in parallel. Secondary antibodies used were goat antirabbit Chromeo 494 (Abcam, UK), Cy3-conjugated streptavidin (Jackson Immunoresearch, USA), goat antimouse Alexa Fluor 488 (Jackson Immunoresearch, USA) and goat antimouse Cy5 (Southern Biotechnology, USA). Immunofluorescence was visualised using a Zeiss LSM 780 Zen Confocal and analysed using Zeiss imaging software (Zeiss, Germany). Five to six 2×2 tile scans were taken from each section at ×400 total magnification. Regions were drawn around tissue; areas with folding or bleeding into tissue caused during biopsy collection/processing were excluded from analysis. As readout, pixels per unit area were calculated.

## Results

### Study participants

Details of study participants used for cytokine mRNA profiling and immunofluorescence studies are shown in tables 1 and 2, respectively. The symptom durations of patients with early arthritis highlight that this population was captured very soon after the onset of their clinically apparent disease (median 5 weeks for those with resolving disease and 6 weeks for those with early RA in the patients who provided samples for cytokine mRNA profiling; median 6 weeks for those with resolving disease and 7 weeks for those with early RA in the patients who provided samples for immunofluorescence studies).

**Table 2:** Demographic and clinical characteristics of study participants used for detection of CXCL4 and CXCL7 by immunofluorescence

	Resolving arthritis	Early RA	Established RA	Early non-RA
Number	9	10	11	5
Symptom duration (weeks); median (IQR)	6 (3–7)	7 (4.8–9.3)	45 (16–53)	7 (2–8.5)
Female; n (%)	3 (33)	5 (50)	6 (55)	2 (40)
Age, years; median (IQR)	37 (33–65)	58 (50–65)	62 (57–65)	41 (37–56)
RF and/or anti-CCP positive; n (%)	0 (0)	4 (40)	6 (55)	1 (20)
*Global disease-related variables*	** **	** **	** **	** **
CRP; median (IQR)	8 (3–13.5)	26 (7.5–58)	10 (0–79)	25 (18.5–54)
ESR; median (IQR)	18 (5.5–51)	19 (4.75–39.25)	50 (34–70)	44 (24–73.5)
DAS28; median (IQR)	4.1 (3.4–5.7)	4.7 (3.8–5.8)	5.2 (4.6–7.5)	4.8 (4.1–6.4)
*Biopsied joint-related variables*				
Joint biopsied				
Ankle; n (%)	2 (22)	2 (20)	3 (27)	2 (40)
Knee; n (%)	7 (78)	7 (70)	8 (73)	3 (60)
MCP joint; n (%)	0 (0)	1 (10)	0 (0)	0 (0)

Early non-RA group: psoriatic arthritis n=2, sarcoidosis n=1, ankylosing spondylitis n=1, unclassified n=1.

CCP, cyclic citrullinated peptide; CRP, C-reactive protein; DAS28, Disease Activity Score in 28 Joints; ESR, erythrocyte sedimentation rate; MCP, metacarpophalangeal; na, not available; RF, rheumatoid factor.

### Synovial cytokine mRNA expression profiles in early arthritis

**Figure 1:**
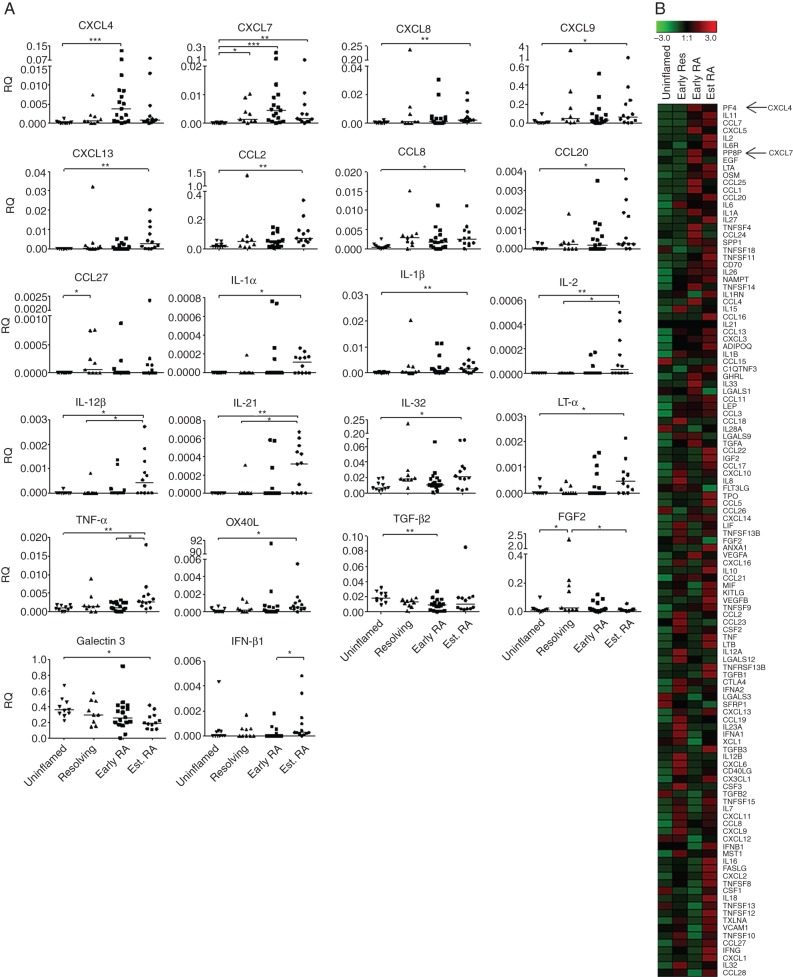
Cytokine and chemokine mRNA expression in synovial biopsies from uninflamed controls and patients with resolving arthritis, early rheumatoid arthritis (RA) and established RA. (A) Synovial tissue sections were assessed from uninflamed controls (n=10) and patients with resolving arthritis (n=9), early RA (n=17) and established RA (n=12). Data for genes for which the Kruskal–Wallis and Dunn's post-test showed significant difference between the four groups are shown. * p<0.05, ** p<0.01, ***p<0.001. (B) Cytokine and chemokine genes were ranked by difference in mRNA expression between resolving arthritis and early RA groups. Means of each group are represented in the heat map. Green represents low and red high relative expression (z-score of mean expression levels).

The mRNA expression of a panel of 117 cytokines and related molecules was assessed in synovial biopsies using low-density real-time PCR arrays. Subject groups investigated were patients with resolving arthritis (n=9), patients with very early RA (n=17), patients with established RA (>3 months’ symptom duration; n=12) and uninflamed control subjects (n=10). Twenty-two genes were identified as being differentially expressed between subject groups by Kruskal–Wallis and Dunn's post-test analysis, shown in figure 1A. Eighteen of these genes were more highly expressed in patients with established RA compared with the uninflamed controls. Of interest, both CXCL4 and CXCL7 mRNA levels were found to be significantly elevated in patients with early RA compared with uninflamed controls, and showed a trend towards higher expression in early RA compared with patients with resolving arthritis. Intriguingly there was also a trend towards higher expression in early RA compared with established RA, suggesting an increase in CXCL4 and CXCL7 levels in the early phase of disease in patients whose arthritis persisted versus those whose arthritis resolved. No significant differences were observed in CXCL4 and CXCL7 expression between cyclic citrullinated peptide (CCP)-positive and CCP-negative patients in either the early RA or established RA groups (see online supplementary figure S2). When cytokine and chemokine mRNA expression was ranked by their difference in expression between resolving arthritis and early RA groups, CXCL4 and CXCL7 were ranked highly in showing differential expression between the two groups (figure 1B).

### Machine learning classification selection of differentiating cytokines and chemokines

**Figure 2:**
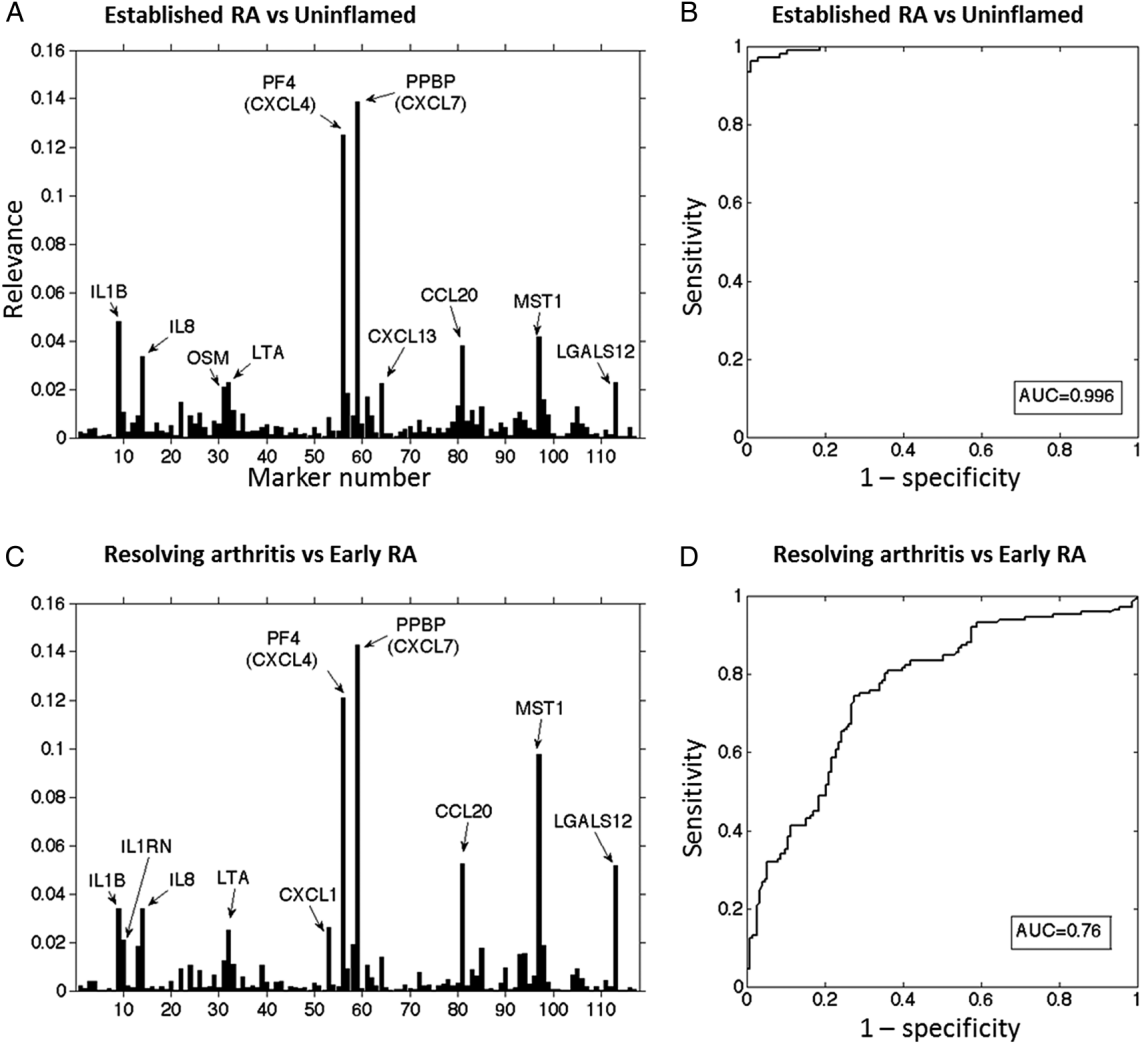
Generalised matrix relevance learning vector quantisation-based discrimination of subject groups (A) All 117 cytokine/chemokine genes used to classify uninflamed controls versus patients with established rheumatoid arthritis (RA). The 10 genes most informative in discriminating groups are indicated. (B) Receiver-operating characteristic (ROC) characteristics of the obtained classifiers for uninflamed controls and patients with established RA. (C) Classification cytokine mRNA signals in resolving arthritis versus patients with early RA. The 10 genes most informative in discriminating groups are indicated. (D) ROC characteristics of the obtained classifiers for patients with resolving arthritis and early RA. AUC, area under the curve.

We applied a novel strategy of multivariate analysis to test whether combinations of gene expression signals rather than individual cytokine mRNA signals could distinguish synovium from patients with self-limiting arthritis from those with early-stage RA. Matrix relevance GMLVQ analysis was applied to the cytokine mRNA data to achieve classification of samples by determining a discriminative distance measure that characterises differences between subject groups. For classification of the established RA and uninflamed groups, analysis of the obtained relevance matrix revealed that the 10 most informative genes in discriminating patients with established RA from uninflamed controls were CXCL7, CXCL4, IL1B, MST1, CCL20, IL8, LGALS12, LTA, CXCL13 and OSM (figure 2A). Receiver-operating characteristic (ROC) analysis was used to assess the performance of classifier models for group classification. For the established RA and uninflamed group classification, GMLVQ yielded an area under the curve (AUC) of 0.996 (figure 2B). For classification of the early RA and resolving arthritis groups, the 10 most informative genes corresponded to CXCL7, CXCL4, MST1, CCL20, LGALS12, IL8, IL1B, CXCL1, LTA and IL1RN (figure 2C). ROC analysis yielded an AUC of 0.764 (figure 2D). In both cases, CXCL4 and CXCL7 played dominant roles in terms of discriminative power.

### CXCL4 and CXLC7 protein expression in synovial tissue

**Figure 3:**
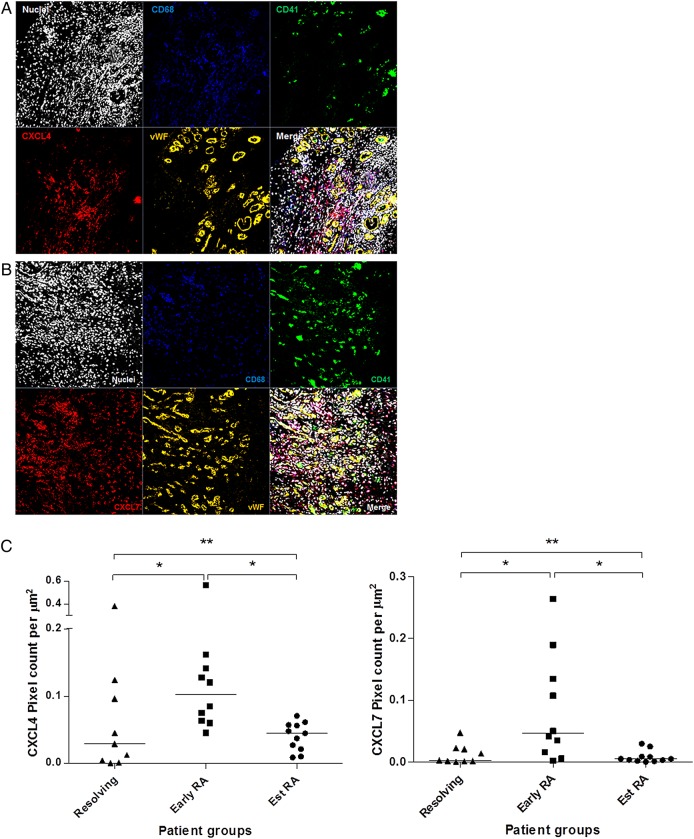
Immunofluorescence staining of CXLC4 and CXCL7 in synovial tissue sections. (A) Synovial tissue staining of CXCL4 (red), CD68 (blue), CD41 (green) and von Willebrand factor (vWF) (orange). (B) Synovial tissue staining of CXCL7 (red), CD68 (blue), CD41 (green) and vWF (orange). Nuclear counterstain is shown. Images are representative of early rheumatoid arthritis (RA) synovium (n=10). No staining was observed using isotype and concentration-matched negative controls. Images were taken at ×40 magnification. (C) Quantification of CXCL4 and CXCL7 staining, calculated as the number of pixels per μm^2^ over 6× 2×2 tile scans at ×40 magnification, in synovial tissue sections from patients with resolving arthritis (n=9), early RA (n=10) and established RA (n=11). Patients with early RA showed a significantly higher level of CXCL4 (p<0.05) and CXCL7 (p<0.05) compared with patients with resolving arthritis and established RA. Kruskal–Wallis and Dunn's post-test; * p<0.05, ** p<0.01.

Since cytokine mRNA profiling suggested an upregulation of CXCL4 and CXCL7 expression in patients with early RA, we next sought to test whether expression of these chemokines was also elevated at the protein level. CXCL4 and CXCL7 were stained in synovial tissue sections from patients with resolving arthritis (n=9), early RA (n=10) and established RA (n=11), and staining was visualised by immunofluorescence. Representative images of staining of CXCL4 and CXCL7 in the synovium are shown in figure 3A, B. Synovial tissue sections were co-stained for CD68 to identify macrophages, CD41 to identify platelets and vWF to identify vascular endothelial cells. Both CXCL4 and CXCL7 were found to be expressed in the synovium of all subject groups examined. However, expression of both CXCL4 and CXCL7 was significantly elevated in patients with early RA compared with patients with resolving arthritis (CXCL4 and CXCL7; p<0.05) and patients with established RA (CXCL4 and CXCL7; p<0.05; figure 3C), which supported the findings made at the mRNA level. Quantification of protein levels of CD41, which is specifically expressed by platelets, did not show significant differences (data not shown). The groups of patients investigated in the cytokine mRNA profiling and those tested for immunofluorescence studies were partially overlapping. Protein data from seven patients who were not investigated in the mRNA study also showed a higher level of CXCL4 and CXCL7 protein expression in the patients with early RA compared with those with established disease (see online supplementary figure S3A and B). A small number of patients developed chronic inflammatory joint diseases other than RA (table 2). Their CXCL4 and CXCL7 levels are shown in online supplementary figures S3C, D.

**Figure 4:**
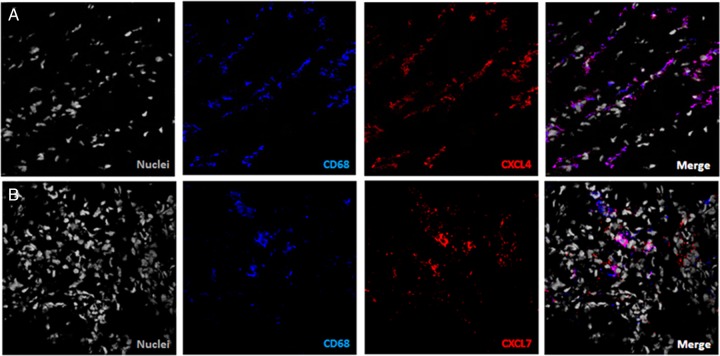
Co-localisation of CXCL4 and CXCL7 with CD68-positive cells. (A) Synovial tissue staining of CXCL4 (red) and CD68 (blue) and (B) staining of CXCL7 (red) and CD68 (blue) showed co-localisation of both cytokines with macrophages. Image representative of rheumatoid arthritis synovium (n=10).

**Figure 5:**
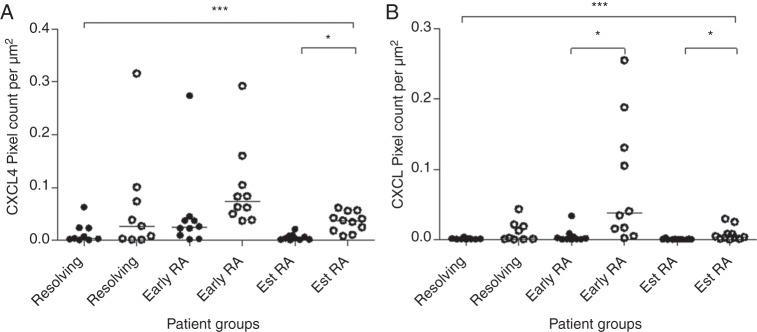
Expression of CXCL4 and CXCL7 in the synovium is predominantly found outside the vasculature. Staining of CXCL4 and CXCL7 in synovial tissue sections was quantified inside and outside of the vasculature, as assessed by co-staining with von Willebrand factor. Expression of both chemokines was significantly elevated outside the vasculature. RA, rheumatoid arthritis. Kruskal–Wallis test, Dunn's post-test; *p<0.05, ***p<0.001.

In the synovium of all subject groups studied, CXCL4 and CXCL7 staining was found to co-localise with CD68 staining, indicating an association of both of these chemokines with macrophages (figure 4A, B). As expected, CXCL4 and CXCL7 staining was also observed on CD41-expressing platelets (figure 3). To further investigate this finding, synovial tissue samples were stained for vWF and expression of CXCL4 and CXCL7 was quantified inside and outside of the vasculature. Quantification of positive pixels showed that CXCL4 and CXCL7 were found predominantly outside the vasculature (figure 5A, B). In addition, the specificity of the CXCL7 staining was confirmed by inhibition of staining with preincubation of the antibody with recombinant CXCL7 (see online supplementary figure S1).

## Discussion

In this study, we have identified two chemokines, CXCL4 and CXCL7, which are expressed in the earliest clinically apparent stage of RA. They were predominantly detected on macrophages infiltrating the synovium, indicating a previously undescribed role for this cell type in contributing to RA pathogenesis in the very early phase of disease.

Macrophages are found in large numbers throughout the rheumatoid synovium. Type A synoviocytes are macrophage-derived cells that constitute part of the healthy synovial lining while large numbers of activated macrophages are found in the inflammatory infiltrate in the sublining and at the pannus–cartilage interface. The origin of these cells is a matter of debate; they could derive from the proliferation of tissue-based macrophages or from circulating monocytes entering from the blood stream.^14–17^ Macrophages are important contributors to inflammation and joint destruction due to their production of proinflammatory mediators and tissue-degrading enzymes. Furthermore, the number of synovial macrophage has been found to correlate with joint erosion in RA while changes in the numbers of CD68+ macrophages correlate with therapeutic success of a range of therapies.^18–20^

CXCL4 is chemotactic for neutrophils, fibroblasts and monocytes, prevents monocyte apoptosis, induces differentiation of monocytes into macrophages and enhances monocyte phagocytosis and oxygen radical production.^21–24^ CXCL7 is involved in neutrophil chemotaxis and activation, and activates connective tissue cells.^25^ The functions described for CXCL4 and CXCL7 suggest that in RA these chemokines could not only exacerbate synovial inflammation but also promote its chronicity by attracting monocytes to the inflamed tissue and activating them following recruitment to the synovium. Our finding that CXCL4 and CXCL7 are highly expressed during the first 12 weeks of synovitis in patients who develop RA but are found at lower levels in longer duration RA may reflect local pathological changes occurring during this critical phase, which has been described as the therapeutic 'window of opportunity'. A similar phenomenon was recently reported by van Bon *et al,*^26^ who demonstrated high levels of CXCL4 expression in patients at an early stage of systemic sclerosis.

Previous studies have suggested roles for CXCL4 and CXCL7 in RA. Elevated levels of CXCL7 have been reported in the serum, synovial fluid and synovial tissue of patients with RA.^27^ ^28^ While CXCL7 promotes angiogenesis, CXCL4 has an antiangiogenic effect.^29^ The elevated expression of the angiostatic chemokine CXCL4 during the early phase of disease may reflect an attempt to prevent or minimise the first signs of angiogenesis that takes place in the RA synovium.

In our study, CXCL4 and CXCL7 expression in the synovium was found to be predominantly localised to CD68+ macrophages, with less co-localisation observed with platelets. Where CXCL4 and CXCL7 expression was seen to co-localise with platelets in the synovium, this was mainly confined to vessel thrombi. The genes for CXCL4 and CXCL7 are located in a gene cluster comprising several CXC chemokines in a locus on chromosome 4,^30^ ^31^ which was once considered to be megakaryocyte-specific. However, other studies have reported that expression of these chemokines is not restricted to the megakaryocyte lineage. A recent study using a PF4-Cre mouse model reported that CXCL4 could be produced by both myeloid and lymphoid lineages, with CXCL4 transcripts detected in adult haematopoietic stem cells.^32^ In a similar reporter gene model, CXCL4 expression was shown in mature murine macrophages.^33^ Furthermore, Schaffner *et al*^34^ reported expression of CXCL4 by human monocytes and found that CXCL4 expression was upregulated upon monocyte activation. Intriguingly, a recent study reported CXCL4 expression by activated T cells that limited Th17 differentiation.^35^ Monocytes have been described to constitutively transcribe and translate CXCL7, which is processed intracellularly into several derivatives known to have signalling and effector functions.^36^ Together this evidence is supportive of our finding that CXCL4 and CXCL7 is associated with and may be produced directly by macrophages in the synovium during very early RA.

Interest in chemokines in RA has been revived by findings such as detection of citrullinated chemokines in RA synovial fluid that have enhanced chemotactic activity^37^ and the prospect of using antichemokine targeting for therapeutic purposes.^38^ ^39^ We have interpreted the high levels of CXCL4 and CXCL7 found in early RA compared with resolving arthritis or established RA to represent a transient upregulation of these cytokines based on a cross-sectional analysis of samples; it would be interesting to confirm the transient nature of this upregulation in a longitudinal study. However, the ethical implications would make an observational study of the evolution of synovial tissue pathology from early to established RA in the absence of therapeutic intervention impossible to conduct in patients. In the future, it will be important to investigate whether the production of CXCL4 and CXCL7 observed in the synovium in early RA is reflected by elevated levels in plasma samples. Future use of these chemokines as biomarkers for prediction of progression to RA will depend on replication in other independent cohorts.

## Supplementary Material

Web supplement

Web figures

Web table
