# Supplementary material for: Expression of chemokines CXCL4 and CXCL7 by synovial macrophages defines an early stage of rheumatoid arthritis
Source: Ann Rheum Dis. 2015 Apr 9;75(4):763–71. doi: 10.1136/annrheumdis-2014-206921 (PMC4819606; doi:10.1136/annrheumdis-2014-206921)
Supplement: Web supplement [file annrheumdis-2014-206921-s1.pdf]

Supplementary text: Machine Learning Analysis

All synovial expression values were log-transformed after replacing zeros by the smallest non-zero expression observed in the data set, i.e. 0.00002. This yielded in total n=47 samples, each of which was represented by a 117-dimensional vector $\mathbf{x}_i$ (i=1,2,…47) of log-transformed excretion values.
In order to reduce the dimensionality, a principal component analysis was performed. Note that projections on 47 principal components would realize a perfect representation of the entire data set, which comprises 47 samples. We observed that the 21 leading principal components already explained 95% of the observed variation in the data set. Assuming that the remaining 5% are most likely dominated by noise, we restricted our analysis to the corresponding 21-dimensional projections, representated by vectors $\mathbf{y}_i = \mathbf{P}\ \mathbf{x}_i$, where $\mathbf{P}$ is the (21x47)-dim. transformation matrix obtained in the PCA. The resulting 47 vectors $\mathbf{y}_i$ were labeled according to the subject groups: uninflamed controls (n=9), early resolvers (n=9), early RA (n=17), and established RA (n=12).
Further computational analysis was carried out with the aim of differentiating (a) between uninflamed controls and established RA and (b) between early resolvers and patients with early RA. Specifically, we employed a variant of Learning Vector Quantization (LVQ) [1] which incorporates the data driven identification of an adaptive distance measure in the training process: Generalized Matrix Relevance LVQ (GMLVQ) [2].
In LVQ, the classification is based on *prototypes*, i.e. typical representatives of the classes, obtained from labeled example data in a computerized training process, which serve as reference vectors to compare observed data with. In so-called *Nearest Prototype Schemes*, data points are assigned to the class represented by the closest prototype, identified in terms of a suitable distance measure. Here, we employed only the simplest setting with a single prototype per class and an adaptive quadratic distance measure. In mathematical terms, sample projections $\mathbf{y}$ and 21-dim. prototypes $\mathbf{w}$ are compared in terms of the quadratic form $d(\mathbf{y},\mathbf{w}) = (\mathbf{y}-\mathbf{w})^T\ \Gamma\ (\mathbf{y}-\mathbf{w}) = (\mathbf{x}-\mathbf{v})^T\ P^T\ \Gamma\ P\ (\mathbf{x}-\mathbf{v})$. Here, $\mathbf{v}$ corresponds to a prototype in the original 117-dim. space of expression values with $\mathbf{w}=P\ \mathbf{v}$.
As discussed in [2], off-diagonal elements Λ_{ij} of the (117x117)-dim. matrix $\Lambda = P^T\ \Gamma\ P$ quantify the weight with which a pair of cytocines i and j contributes to the distance measure. Similarly, the diagonal elements Λ_{ii} can be interpreted as the relevance of a particular cytocine i in the classification scheme.
In each individual training process we applied an additional z-score-transformation such that for all markers the mean of the transformed excretion over the training set was *zero* and the corresponding variance was *one*. A more detailed mathematical description of GMLVQ training can be found in [2]. Essentially, the training process determines the optimal positions of the prototypes and configuration of the matrix Γ in the distance measure. The iterative optimization is guided by a suitable cost function, which evaluates the classifier's performance on the training samples [2]. Here, we used a gradient descent technique with adaptive step size, described in [3], and stopped the training process when the rate of misclassifications in the respective training set was lower than 1% and appeared to be stationary.
Initial values for the prototypes were determined from the class conditional means observed in the training data. In the classification of (a) uninflamed controls vs. established cases of RA, the matrix Γ was initially set to the identity, corresponding to the unbiased a priori assumption of all 21 projections being equally relevant. In the more difficult problem (b) of discriminating early resolvers and patients with early RA, the matrix Γ as obtained in the former problem served as the initial configuration, thus making use of prior knowledge about the relevances from problem (a).
The performance of the trained systems was evaluated following a cross validation concept [4]. In each run of the training process, one sample from each class was excluded from the training set. After training, this pair served as test samples. This procedure was repeated for all possible pairs formed from the two classes under consideration. The test set accuracies achieved by the GMLVQ classifiers were then evaluated on average over all training runs, i.e. over 108 runs for problem (a) and over 153 runs in problem (b). The full Receiver Operating Characteristics (ROC) [5] was obtained by introducing a variable threshold when comparing distances of a data point from the prototypes [6]. Finally, the area under the curve (AUC) was obtained by numerical integration.
In addition, the resulting relevance matrix $\Lambda = P^T\ \Gamma\ P$ was obtained on average over the validation runs. Diagonal elements were inspected in order to identify and compare the most discriminative markers in problems (a) and (b), respectively. A structurally similar application of GMLVQ is presented and explained in greater detail in [6].

References

1. Kohonen, T. (1996) *Self-organizing maps*. Springer, New York, 426 pages.
2. Schneider, P., Biehl, M., Hammer, B. (2009) *Adaptive Relevance Matrices in Learning Vector Quantization.* Neural Computation **21**: 3532-3561.
3. Papari, G., Bunte, K., Biehl, M. (2011) Waypoint averaging and step size control in learning by gradient descent. In: Proc. Mittweida Workshop on Computational Intelligence 2011, Machine Learning Reports MLR-2011-06: 16-26.
4. Duda, R.O., Hart, P.E., Stork, D.G. (2000) *Pattern classification*. Wiley, New York, 654pages.
5. Fawcett, T. (2006) *An introduction to ROC analysis.* Pattern Recognition Letters **27**: 861-874.
6. Biehl, M. , Schneider, P., Smith, D., Stiekema, H., Taylor, A., Hughes, B., Shackleton, C., Stewart, P. Stewart, Arlt, W. (2012) *Matrix Relevance LVQ in steroid metabolomics based classification of adrenal tumors.* In: M. Verleysen (ed.), Proc. 20th Europ. Symp. on Artificial Neural Networks (ESANN 2012), 423-428.
