# Supplementary material for: Expression of chemokines CXCL4 and CXCL7 by synovial macrophages defines an early stage of rheumatoid arthritis
Source: Ann Rheum Dis. 2015 Apr 9;75(4):763–71. doi: 10.1136/annrheumdis-2014-206921 (PMC4819606; doi:10.1136/annrheumdis-2014-206921)

Supplementary Figure 1:
Specificity of the CXCL7 staining was confirmed by inhibition of the staining by pre-incubation of the antibody with recombinant CXCL7. CXCL7 staining shown in red, DNA counterstain shown in blue.

Supplementary Figure 1

Specificity of the CXCL7 staining was confirmed by inhibition of the staining with preincubation of the antibody with recombinant CXCL7

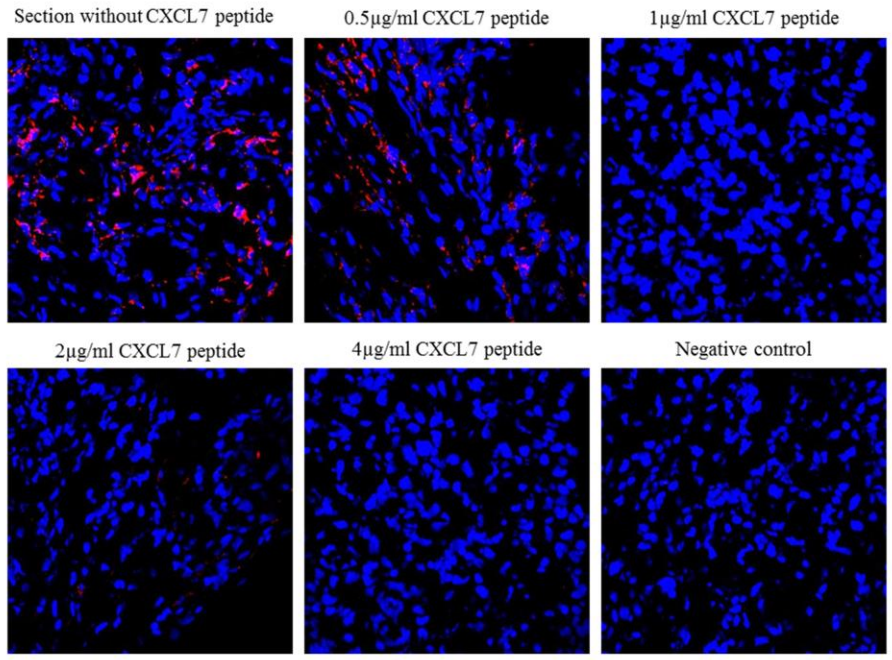

Supplementary Figure 2:
Quantification of CXCL4 and CXCL7 mRNA expression (A and B) and immunofluorescence staining (C and D). In synovial tissue sections from patients with early RA and established RA. Data divided into CCP- (empty symbols) and CCP+ patients (solid symbols). Kruskal-Wallis and Dunn’s post-test. Not significant (NS)

Supplementary Figure 2

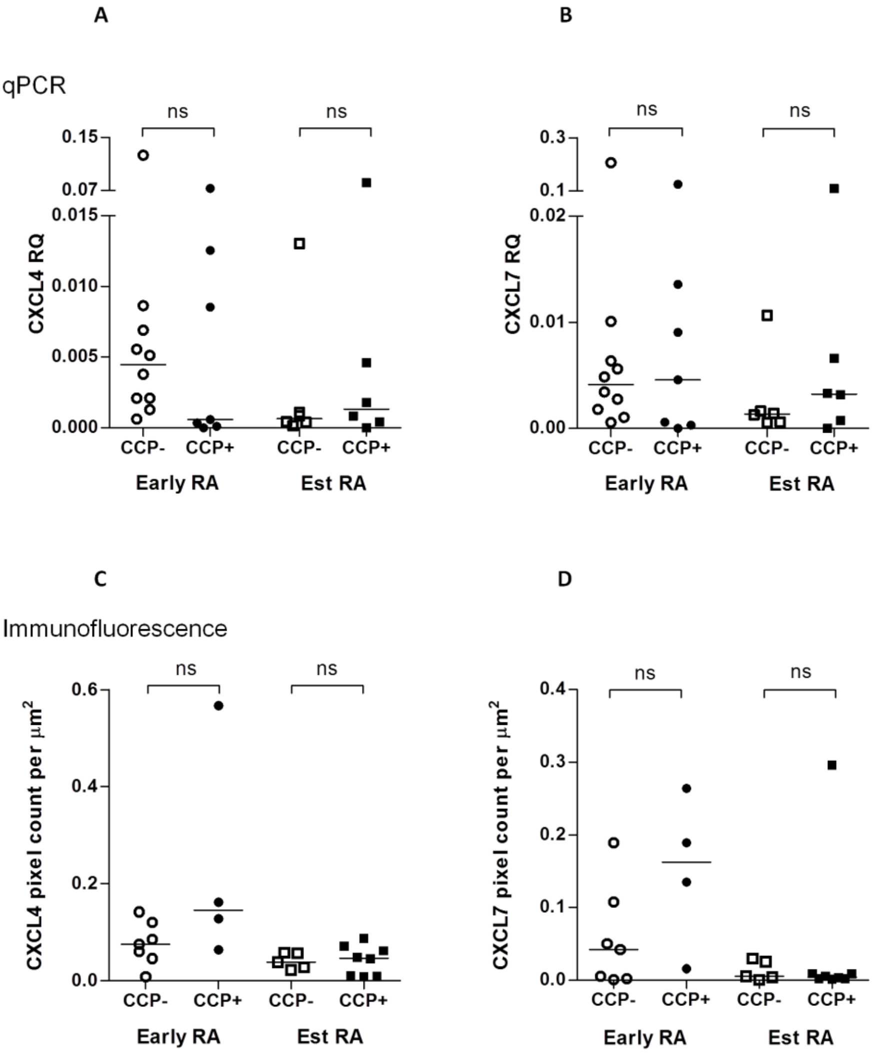

Supplementary Figure 3: Quantification of CXCL4 (A) and CXCL7 (B) immunofluorescence in synovial biopsies from patients not investigated in the mRNA expression study. Quantification of CXCL4 (C) and CXCL7 (D) immunofluorescence in synovial biopsies from patients with other chronic arthritides compared to samples from patients with resolving arthritis, early RA and established RA. Early non-RA group (n=5): psoriatic arthritis n=2 (diamond), sarcoidosis n=1 (inverted triangle), ankylosing spondylitis n=1 (triangle), unclassified n=1 (square). CXCL4: Kruskal-Wallis test * p=0.0160, Dunn's post-test Early RA vs Est RA * p= <0.05. CXCL7: Kruskal-Wallis test **p=0.0081, Dunn's post-test Early RA vs Est RA * p= <0.05, Dunn's post-test Resolving vs Early RA * p=<0.05. CXCL4 and CXCL7: Dunn's post-test Early non-RA vs all other groups = not significant.

Supplementary Figure 3

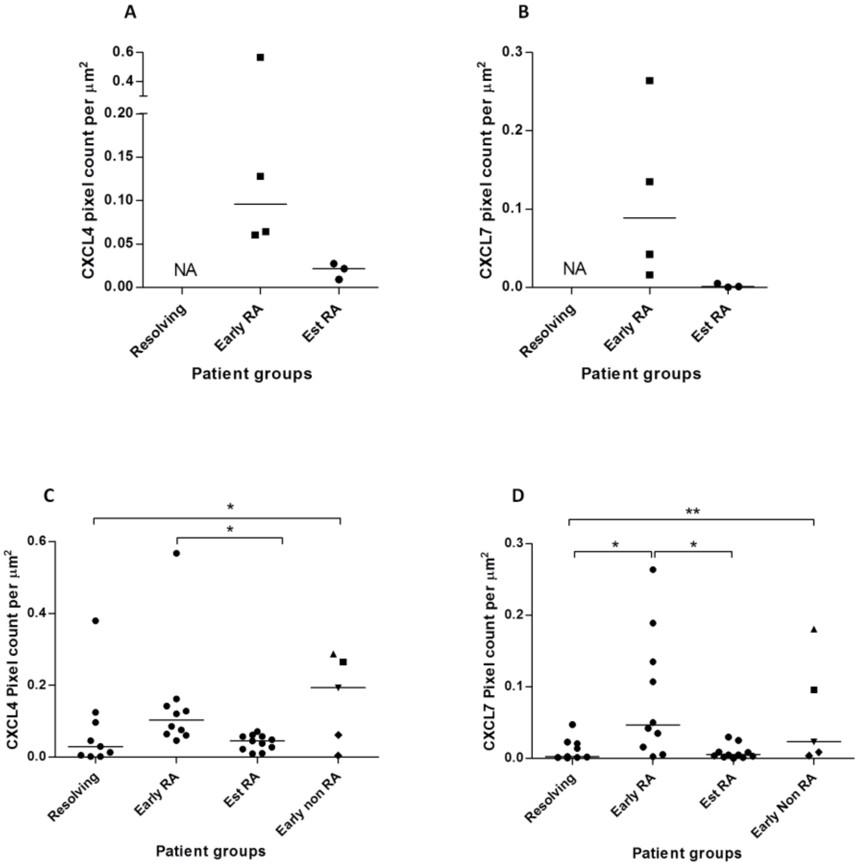

Supplement: Web figures [file annrheumdis-2014-206921-s2.pdf]
