# Supplementary material for: Expression of chemokines CXCL4 and CXCL7 by synovial macrophages defines an early stage of rheumatoid arthritis
Source: Ann Rheum Dis. 2015 Apr 9;75(4):763–71. doi: 10.1136/annrheumdis-2014-206921 (PMC4819606; doi:10.1136/annrheumdis-2014-206921)
Supplement: Web table [file annrheumdis-2014-206921-s3.pdf]

Supplementary Table 1

Custom Gene Set 1

Gene symbol	Gene name
CSF3	Colony-stimulating factor 3
CSF2	Colony-stimulating factor 2
CSF1	Colony-stimulating factor 1
IFNA1	Interferon alpha 1
IFNA2	Interferon alpha 2
IFNB1	Interferon beta 1
IFNG	Interferon gamma
IL1A	Interleukin 1 alpha
IL1B	Interleukin 1 beta
IL1RN	Interleukin 1 receptor antagonist
18S	18S
IL2	Interleukin 2
IL3	Interleukin 3
IL4	Interleukin 4
IL5	Interleukin 5
IL6	Interleukin 6
IL7	Interleukin 7
CXCL8	Chemokine (C-X-C motif) ligand 8
IL9	Interleukin 9
IL10	Interleukin 10
IL11	Interleukin 11
IL12A	Interleukin 12 alpha
IL12B	Interleukin 12 beta
IL13	Interleukin 13
IL14	Interleukin 14
IL15	Interleukin 15
IL16	Interleukin 16
IL17A	Interleukin 17A
IL17F	Interleukin 17F
IL18	Interleukin 18
IL19	Interleukin 19
IL20	Interleukin 20
IL22	Interleukin 22
IL23A	Interleukin 23 alpha
IL24	Interleukin 24
IL25	Interleukin 25
IL26	Interleukin 26
IL27	Interleukin 27
IL28A	Interleukin 28 alpha
IL29	Interleukin 29
IL32	Interleukin 32
IL33	Interleukin 33
LIF	Leukemia inhibitory factor
OSM	Oncostatin M
LTA	Lymphotoxin alpha
TNF	Tumour necrosis factor alpha
LTB	Lymphotoxin beta
OX40L	OX40 ligand
CD40LG	CD40 ligand
FASLG	FAS ligand
CD70	CD70
CD30L	CD30 ligand
4-1BBL	4-1BB ligand
TRAIL	TNF-related apoptosis inducing ligand
RANKL	receptor activator of nuclear factor kappa B ligand
TWEAK	TNF-related WEAK inducer of apoptosis
APRIL	A proliferation-inducing ligand
BAFF	B-cell-activating factor
LIGHT	Ligand for herpes virus entry mediator
TL1A	TNF ligand-related molecule 1
GITRL	Glucocorticoid-induced TNF-related ligand
CD40	CD40
FAS	FAS
GITR	Glucocorticoid-induced TNFR-related protein
TGFB1	Transforming growth factor beta-1
TGFB2	Transforming growth factor beta-2
TGFB3	Transforming growth factor beta-3

Custom Gene Set 2 designed to determine expression of weakly-expressed genes and genes with non-intron spanning primers.

Gene symbol	Gene name
CSF2	Colony-stimulating factor 2
CSF3	Colony-stimulating factor 3
IFNA1	Interferon alpha 1
IFNA2	Interferon alpha 2
IFNB1	Interferon beta 1
IFNG	Interferon gamma
IL1A	Interleukin 1 alpha
IL2	Interleukin 2
IL3	Interleukin 3
IL4	Interleukin 4
IL5	Interleukin 5
IL9	Interleukin 9
IL11	Interleukin 11
IL12A	Interleukin 12 alpha
IL12B	Interleukin 12 beta
IL13	Interleukin 13
IL17A	Interleukin 17A
IL17F	Interleukin 17F
IL19	Interleukin 19
IL20	Interleukin 20
IL21	Interleukin 21
IL22	Interleukin 22
IL23A	Interleukin 23 alpha
IL24	Interleukin 24
IL25	Interleukin 25
IL28A	Interleukin 28 alpha
IL29	Interleukin 29
CCL1	Chemokine (C-C motif) ligand 1
CCL7	Chemokine (C-C motif) ligand 7
CCL11	Chemokine (C-C motif) ligand 11
CCL25	Chemokine (C-C motif) ligand 25
LTA	Lymphotoxin alpha
TL1A	TNF ligand-related molecule 1
KITLG	KIT ligand
ADIPOQ	Adiponectin
MIF	Macrophage migration inhibitory factor
GAPDH	Glyceraldehyde-3-phosphate dehydrogenase
